# 肺癌伴肺裂发育不良应用无肺裂肺叶切除法疗效观察

**DOI:** 10.3779/j.issn.1009-3419.2013.03.06

**Published:** 2013-03-20

**Authors:** 桐 邱, 毅 沈, 栋 王, 滋宗 王, 煜程 魏

**Affiliations:** 266003 青岛，青岛大学医学院附属医院胸外科 Department of Thoracic Surgery, Affiliated Hospital of Medical College, Qingdao University, Qingdao 266003, China

**Keywords:** 肺肿瘤, 无肺裂肺叶切除术, 持续漏气, 胸腔闭式引流, 肺裂发育不良, Lung neoplasms, Fissureless lobectomy, Persistent air leaks, Thoracic close drainage, Fused fissure

## Abstract

**背景与目的:**

肺癌伴肺裂发育不良患者应用传统肺叶切除方法常导致术后发生肺持续漏气。本研究旨在观察无肺裂肺叶切除技术对这类患者的疗效，探讨此技术的临床应用价值。

**方法:**

回顾性分析2011年8月-2012年12月青岛大学医学院附属医院胸外科274例肺癌伴肺裂发育不良的临床资料。按肺叶切除技术分为无肺裂肺叶切除组（A组）和传统肺叶切除组（B组）。采用SPSS 17.0软件处理数据，*Kaplan*-*Meier*法计算术后累计肺漏气停止时间，*Logistic*回归进行多因素分析。

**结果:**

A组较B组的肺持续漏气发生率（*P*=0.009）和术后漏气时间（*P* < 0.001）明显降低；两组术后胸腔引流时间、术后胸腔液体引流量以及术后住院时间差异无明显统计学意义。

**结论:**

肺癌伴肺叶发育不良应用无肺裂肺叶切除技术，能明显降低术后肺持续漏气发生率及术后肺漏气时间，效果确切。

## Abstract (en)

**Background and objective:**

Traditional lobectomy techniques often lead to persistent air leak (PAL) for lung cancer patients companied with fused fissure. The aim of this study is to evaluate the outcomes of those patients undergoing fissureless lobectomy.

**Methods:**

The clinical data were retro-respectively analyzed from 274 lung cancer patients with fused fissure who received surgical procedures from August 2011 to December 2012. Patients were divided into fissureless group (group A, *n*=121) and traditional group (group B, *n*=153) according to the type of lobectomy techniques. The data were analyzed using SPSS 17.0. The air leak cessation was determined using the *Kaplan*-*Meier* method. Multiple risk analysis was developed by *Logistic* regression.

**Results:**

The incidences of PAL in 2 groups were 1.7% and 9.2%, respectively (*P*=0.009). The air leak cessation in group A was significantly lower than that in group B (*P* < 0.001). There was no statistical different between 2 groups in the duration of drainage, amount of fluid and length of hospital stay.

**Conclusion:**

The fissureless lobectomy to fused fissure decreases the incidence of persistent air leak and duration of air leak. This technique should be considered effective to fused fissure lobectomy.

术后肺持续漏气（persistent air leak, PAL）是肺叶切除术后最常见并发症之一，发生率为3%-25%，常造成术后胸腔引流管留置时间延长而导致住院时间延长和住院费用增加^[1-3]^。对肺裂发育不良的患者，术中游离肺裂常增加肺组织的损伤。本研究回顾性分析肺癌伴肺裂发育不良患者的临床资料，对无肺裂肺叶切除术（fissureless lobectomy）与传统肺叶切除术两种手术方式进行比较。

## 1 资料与方法

### 1.1 患者资料

#### 1.1.1 纳入标准

2011年8月-2012年12月在青岛大学医学院附属医院胸外科接受肺叶切除术的肺癌患者，术后随访资料完整。符合下列条件者纳入研究：①术前病理诊断为非小细胞肺癌；②肺癌完全切除（按2005年IASLC肺癌完全性切除手术标准^[4]^），并且对至少3组以上的纵隔淋巴结站进行清扫；③纳入肺裂分级为3级-4级，即部分肺裂融合和全部肺裂融合的肺裂发育不良患者（参照Craig肺裂分级方法^[5]^）；④手术方式为肺叶切除术，包括传统肺叶切除技术和无肺裂肺叶切除技术；⑤按2009版UICC肺癌分期标准^[6]^对术后病理进行分期。

#### 1.1.2 资料收集

入组患者共274例，男性163例，女性111例。年龄48岁-71岁，平均年龄63.8岁±4.9岁，详见表 1。按照是否应用无肺裂肺叶切除法，分为无肺裂肺叶切除组（A组，*n*=121）和传统肺叶切除组（B组，*n*=153），采集以下资料：住院号、年龄、性别，术前FVC、FEV_1_、FEV_1_/FVC、血糖受损情况，手术类型、肺叶切除部位、胸腔粘连情况、一次性切割闭合器使用数量、手术时间、术中出血量、术后肺漏气时间、术后胸腔闭式引流时间、术后住院时间以及术后TNM分期。

**1:** A组与B组患者的基线情况 Characteristics of patient baseline between group A and group B

Characteristic	Group A (*n*=121)	Group B (*n*=153)	*P*
Age (year)	63.7±5.2	64.0±4.8	0.621
Male	73	90	0.801
FVC (L)	2.77±0.63	2.81±0.79	0.659
FEV_1_ (L)	1.99±0.72	2.01±0.75	0.824
FEV_1_/FVC (%)	86.8±13.9	87.4±17.7	0.753
FPG damage	38	46	0.811
Smoker or ex-smoker	65	86	0.681
Right upper lobectomy	65	101	0.039
VATS surgery	92	122	0.461
Strong pleural adhesion	8	11	0.852
Number of staplers	4.2±0.8	3.2±1.0	< 0.001
Operative time (min)	205±43	198±39	0.160
Blood loss (mL)	251±64	264±70	0.114
pTNM			0.914
pⅠa	10	12	
pⅠb	70	96	
pⅡa	24	28	
pⅡb	12	11	
pⅢa	5	6	
PⅣ	0	0	
FPG damage: fasting plasma glucose damage, including diabetes (FPG>7.1 mmol/L) and prediabetes (FPG 6.1 mmol/L-7.0 mmol/L); VATS: video assisted thoracoscopic surgery; pTNM: pathological TNM classification; Group A: fissureless group; Group B: traditional group.			

### 1.2 肺叶切除方法

双腔气管插管，全身麻醉。开胸手术由第5肋间外侧切口进胸；电视胸腔镜手术为2孔操作，主操作孔为腋前线第4或第5肋间3 cm-5 cm切口，副操作孔为腋后线第7肋间，观察孔为腋中线第7或第8肋间。应用一次性切割闭合器械（美国强生公司爱惜龙，Endo GIA）处理支气管、血管及叶裂。术中不应用生物蛋白胶，并且不进行胸膜固定术。所有手术均由一组手术组独立完成。

#### 1.2.1 传统肺叶切除技术

按经典手术技术使用剪刀或电刀游离叶裂，暴露叶间肺动脉。

#### 1.2.2 无肺裂肺叶切除技术

肺叶血管的解剖由肺门进行，不对叶裂进行游离。①右肺上叶操作顺序为静脉、尖前支动脉、后升支动脉、上叶支气管，最后使用Endo GIA闭合切断融合的肺裂。其解剖方向由上向下，左肺上叶同此方法。②肺下叶操作顺序为静脉、下叶支气管、下叶动脉各分支，最后闭合切断融合斜裂。其解剖方向为由下向上。③中叶的切除由肺门开始解剖，依次处理中叶静脉、中叶支气管以及中叶动脉，最后闭合切断融合的水平裂和斜裂。术中照片见图 1。

**1:**
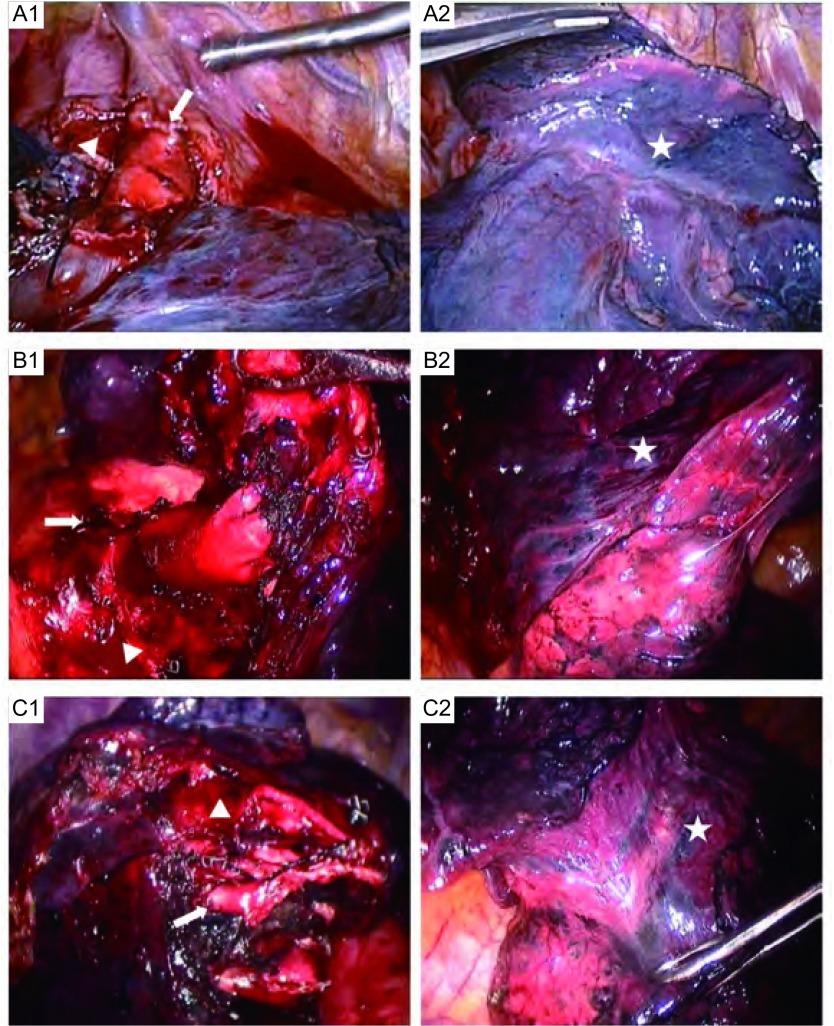
肺癌伴肺裂发育不良行无肺裂肺叶切除术的术中照片。A1：右肺上叶切除处理血管和支气管后照片；A2：使用切割闭合器前的完整右肺肺裂（此图显示肺裂发育尚可）；B1：左肺上叶切除处理血管和支气管后照片；B2：使用切割闭合器前完整左肺肺裂；C1：左肺下叶切除处理血管和支气管后照片；C2：使用切割闭合器前完整的左肺肺裂。图中三角示支气管残端；箭头示动脉残端；五角形示切割闭合前的完整肺裂 Intraoperative pictures of fissureless lobectomy for lung cancer with fused fissure. A1: the photograph of right upper lobectomy after managing the vessels and bronchus; A2: the photograph of complete right fissures before stapling. B1: the photograph of left upper lobectomy after managing the vessels and bronchus; B2: the photograph of complete left fissure before stapling; C1: the photograph of left lower lobectomy after managing the vessels and bronchus; C2: the photograph of complete left fissure before stapling. The triangles show bronchial stumps; the arrows show artery stumps; the pentagons show the complete fissures

### 1.3 术后处理

#### 1.3.1 术后胸腔闭式引流的管理

术后胸腔均留置1枚28号胸腔引流管，上叶切除者置于胸顶，中、下叶切除者置于膈上，连接胸腔闭式引流瓶。术后每6 h观察并记录胸腔引流管液体引流量及漏气情况。术后第1天行床边胸片了解肺复张情况，之后根据病情复查。拔除胸管的标准为患者病情稳定，液体引流量 < 200 mL/d，引流液颜色为淡黄色，无气体引出，复查胸片肺复张良好。

#### 1.3.2 术后肺漏气的定义

肺漏气定义为咳嗽时引流瓶内出现连续气泡溢出。PAL定义为术后肺持续漏气超过5天^[7]^。

### 1.4 统计学方法

所有数据均采用SPSS 17.0统计软件进行处理。计量资料采用Mean±SD表示，采用*t*检验进行组间比较；计数资料采用*χ*^2^检验进行组间比较。术后漏气停止时间采用*Kaplan*-*Meier*法和*Log*-*rank*检验，多因素分析采用*Logistic*回归模型。以*P* < 0.01（双侧检验）为差异有统计学意义。

## 2 结果

### 2.1 两组基线情况比较

所有患者手术过程顺利，无死亡病例。两组患者在年龄、性别，术前FVC、FEV_1_、FEV_1_/FVC、血糖受损情况和术前吸烟情况方面差异无统计学意义。两组患者在切除右肺上叶比率、胸腔镜手术比率、胸腔广泛粘连率、手术时间、术中出血量以及术后TNM分期方面差异无统计学意义。Endo GIA平均钉仓使用数量为3.7个±1个（A组4.2个±0.8个，B组3.2个±1.0个），两组差异有统计学意义（*P* < 0.001）。结果见表 1。

### 2.2 两组术后胸腔引流情况比较

A、B两组术后PAL例数分别为2例和14例，发生率分别为1.7%和9.2%，差异有统计学意义（*P*=0.009）。两组患者在术后胸腔引流时间、术后胸腔引流液体量以及术后住院时间方面差异无统计学意义（表 2）。

**2:** 患者术后胸腔引流情况 Outcome of the postoperative chest drainage

Outcome	Group A (*n*=121)	Group B (*n*=153)	*P*
Postoperative PAL	2	14	0.009
Days of air leaks (d)	1.9±0.5	2.9±0.4	< 0.001^*^
Days of drains (d)	4.2±1.2	4.6±1.4	0.015
Amount of fluid (mL)	1274±325	1328±280	0.130
Length hospital stay (d)	5.4±1.2	5.9±1.8	0.012
^*^Days of air leaks after surgery is estimated by *Log*-*rank* test, *P* < 0.001 by *Log*-*rank* test. PAL: persistent air leak.			

### 2.3 术后肺漏气时间的影响因素

应用*Kaplan*-*Meier*法以及*Log*-*rank*检验进行单因素分析显示，A组术后肺漏气停止时间明显低于B组（*P* < 0.010），肺切除方法是影响术后肺漏气停止时间的重要因素（图 2）。*Logistic*多因素回归模型显示：肺切除方法（95%CI: 0.004-0.378, *P*=0.006）、术前FEV_1_/FVC < 70%（95%CI: 4.343-383.886, *P*=0.001）是影响术后PAL的重要因素。

**2:**
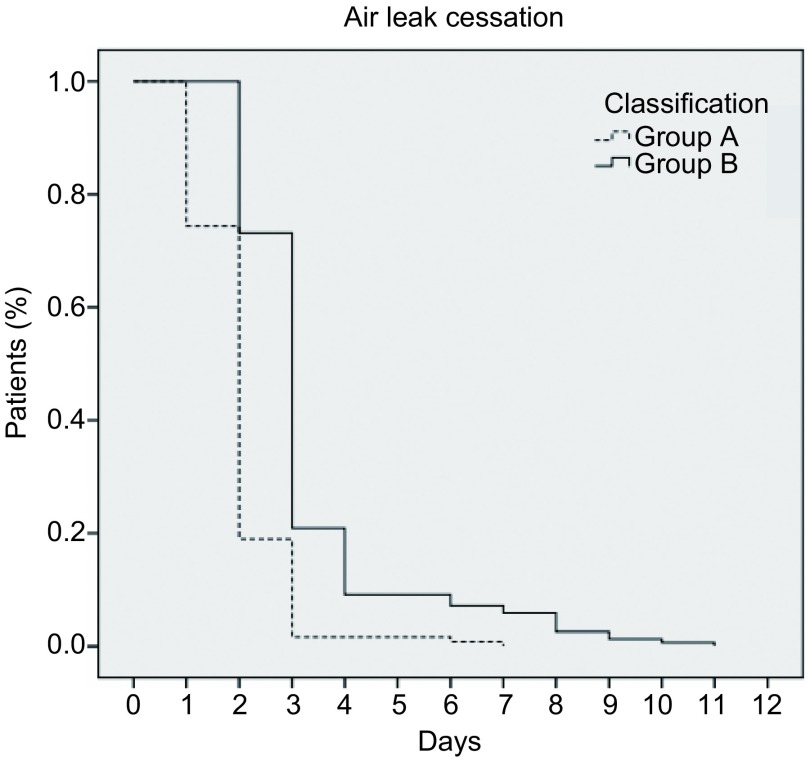
无肺裂肺叶切除组（A组）和传统肺叶切除组（B组）术后*Kaplan*-*Meier*累计肺漏气停止时间曲线 Probability of air leak cessation by *Kaplan*-*Meier* method

## 3 讨论

PAL目前仍是肺切除手术后的常见并发症之一，国际推荐的定义为肺叶切除术后肺持续漏气超过5天^[7]^。目前预防PAL的常用方法有：术前肺保护、术中应用生物蛋白胶或胸膜固定术，术后持续负压吸引等。但是涉及肺裂发育不良的相关报道十分少见。Ciaig^[5]^根据肺裂的融合程度，将肺的叶裂分为4个等级，其中3级-4级可定义为肺裂发育不良，本研究肺裂发育的分级由3名手术医师共同确定。无肺裂肺叶切除技术由Cerfolio等^[8, 9]^首先提出，该方法强调肺叶切除时不经融合肺裂解剖肺动脉，肺裂的切开闭合全部采用机械方法，提示对于肺裂发育不良的患者，可以采用此技术进行肺叶切除。

本研究发现，在平均闭合器使用数量方面两组有明显差异（A组4.2个±0.8个，B组3.2个±1.0个，*P* < 0.001）。回顾手术过程，传统手术方法游离肺裂的范围大，接近切割闭合器钉仓的长度，因此传统手术方法在闭合器使用数量上要优于无肺裂切除组。

国外文献^[10-12]^报道，无肺裂肺叶切除技术能减少术后胸腔闭式引流时间及术后住院时间，此结论与本研究结果不同。对术后漏气停止时间分析发现，80%的患者于术后第3天停止漏气，而此时胸腔液体引流量仍在300 mL/d-350 mL/d，因此术后胸腔闭式引流时间及术后住院时间受胸腔引流液量的影响较大。本研究中，单因素分析显示肺切除方法是影响术后肺漏气时间的重要因素，多因素分析显示肺切除方法、术前FEV_1_/FVC < 70%是影响术后PAL的重要因素。有研究^[13, 14]^指出，除术前合并COPD外，糖尿病、右上叶切除、胸腔粘连是PAL发生的重要因素，但在多因素分析中并未出现统计学差异，故尚待进一步扩大样本深入研究。此外，应用*Kaplan*-*Meier*法分析累计肺漏气停止时间显示，两组的生存曲线未出现明显分离（图 2），提示肺漏气的时间可能与术中因素有关，值得进一步研究。

多项研究^[2, 11, 15-17]^显示，不论胸腔镜或开胸手术，无肺裂肺叶切除加肺门纵隔淋巴结清扫术在术后并发症发生率及肿瘤学预后方面无明显差异。本研究中，除PAL外，A组未见其它术后并发症发生，B组中1例出现PAL伴发纵隔气肿。临床实践中体会，开放手术时，上叶进行无肺裂肺叶切除术相对下叶容易。主要原因是，下叶的解剖方向是由下向上，第5肋间切口相对偏高，而如果降低切口，在淋巴结清扫时视野暴露又不满意。由于电视胸腔镜无视野死角的特点，胸腔镜下的无肺裂切除无此缺陷，配合此技术使手术操作更加安全高效。无肺裂肺叶切除技术减少了术中对肺的反复拨转、牵拉与钳夹，避免了更多的肺组织损伤，有助于术后快速康复，更符合真正意义上的围手术期微创外科理念。需要承认，任何一种手术方法都有缺陷，无肺裂切除法在闭合叶裂前应注意保护其周围的非切除肺的血管和支气管，避免误伤。另外，在遇到血管变异或血管、支气管周围淋巴结粘连致密时，不应强求此技术。

综上所述，对肺裂发育不良的肺癌病例，术中应用无肺裂肺叶切除技术，可明显降低术后漏气时间及术后持续漏气发生率，效果确切。本研究尚存在不足之处，如应用此技术的时间尚短，入组病例数量受限，肺血管的解剖变异未纳入分析等，因此仍需更进一步的研究。
